# Supplementary material for: Delivering culturally adapted family interventions for people with schizophrenia in Indonesia: A feasibility randomised controlled trial and nested process evaluation
Source: PLoS One. 2026 Jan 8;21(1):e0338371. doi: 10.1371/journal.pone.0338371 (PMC12782378; doi:10.1371/journal.pone.0338371)
Supplement: S2 File — (DOCX) [file pone.0338371.s002.docx]

**Research Protocol**

Title: Reducing Relapse for People with Schizophrenia in Indonesia: Developing a culturally-relevant, evidence-based Family Intervention

Principal Investigators: Laoise Renwick and Herni Susanti

Other Investigators: Budi Anna Keliat, Helen Brooks, Penny Bee, Karina Lovell, Timothy Bradshaw

Institutional affiliations: University of Indonesia, University of Manchester

**I. BACKGROUND AND SIGNIFICANCE**

A. Schizophrenia, the most common psychotic illness, is among the ten most disabling conditions worldwide and those in low-and middle income countries (LMIC) are disproportionately affected. Schizophrenia is a complex illness characterised by marked heterogeneity in presentation, course and outcome. Most experience intermittent relapse, impaired functioning in between episodes and the majority experience comorbidity with other mental and physical illnesses most often including depression and anxiety, drug and alcohol abuse, cardiovascular disorders, infectious diseases, nutritional and metabolic diseases and obesity-related cancers. In LMICs up to 90% of people with schizophrenia remain untreated. Consequently, much of the burden of care falls to families and communities without adequate, skills, knowledge or resources to implement care.

B. Effective packages of care for schizophrenia comprise both pharmacological treatments and psychosocial interventions. It is broadly considered that in LMICs, a narrower group of interventions will be feasible due to lack of finance and infrastructure, population density and under-developed social welfare systems. FIps have exceptionally robust evidence for their efficacy in high resource settings and have an emerging evidence base in LMICs. Providing these interventions can reduce relapse, improve the family environment and therapeutic alliances with healthcare workers. The World Bank’s recently published third edition of global disease priorities (DCP3) includes FIps as one of only three potentially cost-effective interventions for people with schizophrenia and recommend these interventions should be prioritised in LMICs.

FIps are theoretically advanced in terms of identifying context-specific needs and the key intervention features that address these. FIps offer therapeutic elements to enhance family skills and knowledge, communication skills training, enhancing skills in problem-solving and goal setting. These are underpinned by theoretical frameworks such as cognitive models, cognitive-behavioural theories and are framed by collaborative partnerships to enhance supportive therapeutic relationships. FIps do require adaptation to different cultural contexts allowing cultural beliefs, explanatory models of illness and contextual socio-economic issues to be incorporated into the content and delivery of such interventions. Indeed, when effective interventions are successfully adapted, people are more likely to engage with psychiatric help offered increasing acceptability. Interventions within specific cultural groups delivered in their native language are twice as efficacious as those delivered without adaptation and cultural adaptation enhances interventions efficacy for treating schizophrenia, the degree of adaptation closely correlated with the degree of efficacy.

**II. STUDY OBJECTIVE(S); INCLUDING SPECIFIC AIMS AND/OR HYPOTHESES**

A. The aim of this 24-month project is to adapt and refine an evidence-based, family intervention for relatives and carers of people with schizophrenia in Jakarta, Indonesia and evaluate the feasibility and acceptability of implementation in primary care settings. Using the Medical Research Council framework for complex interventions we will conduct a three-phase study combining stakeholder consultation and consensus workshops to produce a manual to guide intervention implementation. We will train healthcare workers to deliver the intervention and assess the feasibility and acceptability of conducting a randomised, single-blind trial of our co-produced, culturally-relevant, evidence-based intervention to reduce relapse when compared with standard care.

The study objectives are to:

I. Adapt and refine an evidence-based family intervention using stakeholder preferences in an empirically-derived heuristic framework

II. Co- produce a manual to support the delivery of the culturally-relevant, adapted intervention

III. Identify training needs for healthcare workers and train healthcare workers to deliver the intervention

IV. Evaluate the feasibility of conducting a full trial to test the effectiveness of our intervention and explore the acceptability of the intervention

**III. METHODS**

1. Study Design

The study combines qualitative and consensus methods to develop the intervention and quantitative feasibility study to evaluate delivery. Qualitative methods will be used simultaneously throughout the feasibility trial to evaluate acceptability, tolerability of the intervention and research methods, usability of the intervention resources, adoption of the intervention in primary care and willingness of participants to be randomised.

**Phase 1 stakeholder consultation** aims to determine stakeholder perspectives on how to culturally adapt the family intervention model of Barrowclough and Tarrier to this population. We will convene up to 4 mixed and single participant stakeholder consultation groups with people with schizophrenia, their caregivers and healthcare professionals (mental health and non-mental health trained). We will purposively sample people with SCZ (10-15), caregivers (10-15), healthcare professionals (10-15) based on gender, age, geographical setting and service attendance. We will structure groups based on an existing heuristic model of adaptation including concepts and illness models incorporating culture-specific belief systems and alternatives to the biopsychosocial model, family structures and processes, appropriate communication methods, activities and scenarios to incorporate cultural norms in religious and spiritual practices and adaptations to facilitate feasibility and the development of appropriate therapeutic alliances. Additionally, we will seek suggestions to enhance the cultural validity of the intervention, understand preferences for delivery including session location and duration, therapist preference, suggestions for resources that would support delivery and expectations for therapeutic aims and outcomes based on a heuristic framework for cultural adaptation.

Simultaneously we will conduct qualitative interviews with key informants (n = 10) including service managers, commissioners, ministry officials and community leaders to explore the wider implications of intervention implementation and evaluate factors affecting reach, adoption and maintenance of interventions in primary care settings and the charity sector.

**Phase 2 consensus workshops** will be conducted to gain consensus on the intervention contents, delivery format and training needs for healthcare professionals. We will include 20 experts meaning experts by experience (carers, family members, service users) and experts by profession (academics, health professionals, and service managers) and utilise a modified nominal group technique to decide upon the essential elements of the intervention. We will use co-production principles to generate methods for delivering teaching and training in a ‘train the trainers’ model. The manualised intervention will be developed by the study team, synthesising information from the stakeholder consultation using the heuristic framework into an existing evidence-based family intervention.

**Phase 3 feasibility study** will evaluate the feasibility and acceptability of delivering the intervention in primary care settings in Java, Indonesia. We will test implementation in a single-blind, randomised controlled trial comparing treatment as usual (n = 30) to culturally-adapted family intervention. Over 12 months, we will recruit a convenience sample of 60 service-user and family member dyads to i) compare recruitment and retention in different settings and delivered by different health professionals, ii) assess the feasibility of collecting participant outcome measures at study entry, post-intervention and six months later, iii) assess fidelity to and usability of the intervention manual and evaluate health professionals acceptance of the intervention model and iv) assess the acceptability of the intervention to recipients.

B. Study Population

*Phase 1 Study Population: Stakeholders*

The study populations comprises both individuals with schizophrenia (adult age) and their carers who may be parents, siblings, grandparents, adult-age children. People with schizophrenia will be included if attending a primary health care centre in the sites recruited (Jakarta and Bogor) for assessment and monitoring of symptoms of schizophrenia consistent with DSM-V diagnostic criteria. We will include the carers of people with schizophrenia who provide consent for us to approach for involvement. Key informants will comprise service managers, commissioners, ministry officials and community leaders who have an interest in developing mental health services.

Carers will be included if they are

1. Living with or spending at least 10 hours per week in face to face contact with an individual with schizophrenia and assuming a caring role.

2. Carers/Relatives over the age of 18 years.

3. Resident of Jakarta or Bogor

4. Able to give informed written consent

We will exclude potential participants if they have

1. A relative with drug or alcohol dependence alongside a diagnosis of schizophrenia, according to DSM-V criteria.

2. Unstable residential arrangements such that the likelihood of being available for the duration of the trial is low.

People with psychosis will be included if they

1. Have a diagnosis of schizophrenia or related psychosis and are currently receiving treatment in a primary care setting.

2. Are over the age of 18 years.

3. Resident of Jakarta or Bogor

4. Able to give informed written consent as judged by the referring healthcare worker

*Phase 2 Study Population*

The study population of phase 2 comprises 20 experts by experience (carers, family members, service users) and experts by profession (academics, health professionals, and service managers). We will include people with direct experience of psychosis, those with direct experience of caring for someone with psychosis, academic experts in psychosis research, health professionals in primary and secondary services working with people with psychosis and primary care service managers and directors to contribute to decisions on mode of delivery and content alongside components of the manualised intervention that will support training healthcare workers. We aim to recruit a wide a range of professions with a wide range of expertise including nurses, occupational therapists (OTs), psychologists, psychiatrists and social workers. We will also recruit community workers who bridge the gap between healthcare and communities.

*Phase 3 Study Population*

This phase will include primarily people with psychosis and their family members attending the experimental intervention (anticipated n = 60 dyads, N = 120). We will also conduct process evaluation therefore, a sample of healthcare workers delivering the intervention and those receiving the intervention will be interviewed for their views on the acceptability of the intervention.

C. Assessment of Resources

Four research assistants will work under the direction of investigators in Indonesia (HS & BAK) and will be supported by a project secretary for the duration of the study. The project secretary will also support the Research Advisory Group (RAG) established to support the study processes. For phase 3, trial therapists will comprise healthcare workers in primary care centres whom have already pledged support to the study. Healthcare workers will attend training, deliver all aspects of the intervention, and participate in process evaluation to inform feasibility testing. We have undertaken stakeholder workshops with healthcare workers in primary care centres at district level in Bogor and Jakarta (August 2019) securing their agreement to support the study. Our charity partners (KPSI) in Jakarta have pledged support for recruitment and we will attempt to recruit from this source as a contingency plan if we do not secure sufficient participants from our collaborations with clinical sites.

Training for research assistants in research methods will be provided jointly by members of the UK study team and the Indonesian study team during transitions at each phase of the study (training phases see Gantt chart). Research assistants will hold a degree in nursing and will be working towards a Masters in Nursing Science. Training for the intervention, once manualised, will be provided by our family intervention expert among the study team (TB) supported by the UK and Indonesian study team. Trial therapists will comprise healthcare workers employed in primary care centres.

D. Study Procedures

Include a description of the study procedures (as they relate to the subject). Be sure to include (as applicable):

*Phase 1 Procedures:*

We will conduct a series of four single participant and stakeholder consultation groups comprising people with schizophrenia (n = 10-15), caregivers (n = 10-15) and healthcare professionals from primary care settings (n = 10). Two groups will comprise carers and will be recruited from sites in Bogor and Jakarta. We will purposively sample from each stakeholder group, based on gender, age, geographical setting and service attendance in primary care centres in Jakarta and Bogor. Primary care centres at district level will be recruited to adopt the study from existing networks from stakeholder engagement and the study is supported by the Clinical Lead with responsibility for delivering the government’s new mental health programme in Jakarta from whom we will gain assistance in facilitating access. We will recruit a minimum of 2 primary care centres in each site: Jakarta and Bogor. Primary care centres at district level (*puskesmas*) are typically led by a general physician and will have between 1 and 2 mental health workers delivering the mental health programme.

Service-user and carer participants will be recruited via their mental healthcare workers in *puskesmas* or via self-referral in response to our advertisements. Posters and flyers advertising the study will be placed in appropriate locations in *puskesmas* in Bogor and Jakarta such as visiting/relatives’ rooms and noticeboards and community settings such as community centres and carer support groups. Healthcare professionals will similarly be recruited from these settings but will be informed about the study and inclusion criteria via brief information sessions provided online by the research assistant in each area so they can participate and act as a point of reference for interested service-users and carers.

Potential participants who express an interest and are capable of consenting (as advised by healthcare workers) will have their contact details forwarded to the research team. Potential participants will be identified by healthcare professionals who (having attended an information session about the study delivered online) will approach the potential participant to give details about the study. They will be asked if they are happy for their contact details to be passed to the researcher who will contact the potential participant no sooner than 48 hours after sending a recruitment pack with further information about the study. The initial telephone contact serves as an opportunity to 1. Answer questions the participant may have 2. Check eligibility and 3. Seek consent for involvement. A secondtelephone or online meeting will be arranged if eligible to further discuss involvement in the study and seek consent for involvement. Consent will be digitised and managed in electronic consent management software. Participants email addresses will be obtained and a link will be sent to them where they can view further information about participation and indicate their consent to be involved. At this stage the researcher will also provide tailored guidance on how to use Zoom to enable participation in the consultation groups online and answer any questions. The recruitment pack will contain a participant information sheet, a consent form and a Zoom participation guide. Participants will then be invited to one of four consultation groups delivered online designed to consult stakeholders about content and delivery of a culturally adapted family intervention.

Appendix 1: Participant Information Sheet: Phase 1 (1a, 1b and 1c)

Appendix 2: Consent Form: Phase 1

The consultation guide was developed based on discussions within the research team and our evidence-based intervention and heuristic model from systematic reviews. The guide will be reviewed by the research team for face and content validity and following translation to Bahasa prior to commencing the study and we will pilot the tool on a minimum of 2 participants, optimising the guide ahead of data collection. Consultation groups will be conducted online using video conferencing software i.e. ZoomEach consultation group will be facilitated by two researchers to include two of the following; the Indonesian co-principal investigator (HS), academic early career researchers at UI or a research assistant. Initially, the lead facilitator will outline the study background, purpose of the consultation and session content and process. Each consultation group, with approximately ten participants each, will take approximately 2 hours to conduct and will be audio-recorded for analysis at a later stage. Including time for a break participants will be asked sequentially; experiences of accessing and attending services, perceptions of psychological therapies, impact of illness on families and carers, suggestions to enhance the cultural validity of the intervention, preference for delivery including session duration and location, therapist preference, suggestions for resources that would support delivery and expectations for therapeutic aims and outcomes based on a heuristic framework for cultural adaptation. Participants will be presented an evidence-based model of family interventions and asked to comment on feasibility of engagement, motivation to attend, ways to modify the intervention to engage and retain people with schizophrenia and their families in the intervention. Participants will be reimbursed for travel and subsistence will be provided. This information will be provided to participants in advance of the online consultation meeting to facilitate full participation.

Appendix 3: Consultation Group Topic Guide

Appendix 4: Distress Protocol

We will conduct qualitative interviews with key contributors with specialist roles (n=10) among groups relevant to the project because of their first-hand knowledge of implementing mental health interventions in Indonesia. We will include primary care service managers, commissioners of health services, ministry officials and community leaders who may be able to provide information on the potential impact of the proposed intervention. Key contributors are those who have an intimate knowledge of mental health service delivery in primary care settings in Jakarta and Bogor.

We will use a two-pronged approach to identify potential interview participants among key informants. We will use snowball sampling to generate a sufficient sample of key informants. We will initially approach the lead for the mental health programme in Jakarta and ask to provide basic contact details for people it would be important for us to include in this research and a counterpart in Bogor. We will utilise existing networks and key contacts which we have generated through the development phase of this work and preparation for further collaboration (Collaborative Network Workshops July 2019 & Stakeholder Consultation August 2019) including the head of mental health research in the Ministry of Health and Professor of Nursing at UI with wide networks in clinical settings. We will create a list of potential participants through our engagement with these contacts and networks. To maintain equitable representation, potential key informants will be systematically categorized as academic, government, organization/association, and healthcare/clinical representatives and will be allocated based on location in either Bogor or Jakarta. Five potential informants will be chosen from each area and representing each category before approaching additional potential informants to participate. We will also use snowball sampling and at the end of each interview we will ask the participant to identify any other key informants that would be relevant to include. This will be an iterative process until the number of key informants is 12 participants. Potential participants will be contacted directly by e-mail or via e-mail introductions from others.

Appendix 5: Key Informant Guide

The interview guide was developed based on discussions within the research team and our evidence-based intervention and heuristic model from systematic reviews. The guide will be reviewed by the research team for face and content validity and following translation to Bahasa prior to commencing the study. The interview guide is designed to facilitate interviews lasting 30–60 minutes exploring perceptions of need and mental health service delivery, perceptions of psychosocial therapies and factors that affect the implementation of these in primary care. The guide also explores factors affecting the potential adoption and reach of the intervention, if adopted. Interviewers will also explore factors that influence the smooth running of the feasibility trial and the potential for full-scale trial development. Interviews will be audio-recorded in online software (Zoom) for analysis, and telephone interviews may be used in circumstances where online face-to-face interviews are a challenge to arrange thus interviewer observations will be the primary method of data collection. As before, data will be stored in accordance with the Research Data Management Plan.

*Evidence Synthesis for Intervention Development*

Using empirical findings from the consultation group and the key informant interviews and an empirically-derived heuristic framework for adapting psychosocial interventions, we will develop an evidence matrix tabulating our findings. The matrix will detail points of commonality, divergence and represent key ideas about the content and delivery of the intervention, barriers and facilitators to delivery and engagement supplemented with points about adoption and implementation. Evidence synthesis will be conducted collaboratively by Indonesian and UK researchers during preparatory discussions on Zoom prior to collaborative meetings and the consensus workshops.

*Phase 2 Procedures:*

A formal consensus development process will be undertaken by the researchers based on a modified nominal group technique (NGT). Members of the research team (HS & B.K) will invite 20 experts by experience to attend two workshops from existing networks. Participants will be purposively sampled to reflect specialist knowledge and experience in psychosis care as service recipients and healthcare professionals, education and research. Invited participants will represent key stakeholders including service-users, family members, healthcare workers, community leaders and cadres and academic researchers from wider groups. Not all experts will attend both workshops. More PPI and healthcare participants will be invited to the consensus workshop on day 1 and day 2 will comprise a greater number of educators, academics and researchers consistent with the objectives of each workshop. The technique will comprise discussion, item generation and voting in rounds and at different stages to achieve two aims; decide upon the components of the intervention and generate ideas for training therapists to deliver the intervention. NGT used in this way will define the key components of a culturally-relevant family intervention for people with schizophrenia using a structured, stepwise process in the first workshop. The second workshop will be used to generate resources for delivering and training healthcare workers to administer the intervention. NGT groups allow for participants to discuss recommendations face to face and can maximize the chances for all participants to contribute in an equal way.

Once ‘experts’ agree to attend, the researcher will send a recruitment pack including the rationale for the study and a brief review of the evidence matrix alongside a participant information sheet and consent form. A further follow-up phone call from the researcher will give participants the opportunity to discuss participation, ask questions and to inform them of details about the workshops. Participants will then be invited to attend one of two workshops or both depending on their specific expertise. Each workshop will be conducted online via Zoom.

Participant information sheets and consent forms are provided below.

Appendix 6: Participant Information Sheet: Phase 2 (Appendix 6a, 6b, 6c, 6d)

Appendix 7: Consent Form: Phase 2 (Appendix 7a, 7b)

*Day 1: Expert Consensus Meeting*

Firstly, we will open the workshops with a brief introduction to the study and each other and present the findings from the evidence matrix to the participants. This information will also be provided in advance of the workshop. A series of questions will be presented in PowerPoint representing the key areas of disagreement among data obtained at phase 1. Participants will vote in private on their preferred option and on the next screen provide a short statement explaining this to the group. At this point, individual opinions are still anonymous. Following a break where the facilitators will briefly collate information, the group will be asked to separate into small groups of 4-6 using Zoom break-out rooms and they will be presented with key areas of divergence that remain following voting and each group will be asked to generate consensus and feed back to the remaining groups. This iterative process will continue until consensus is achieved.

*Day 2: Resource and Training Development*

On the second day, we will introduce participants to familiarise themselves again with the planned intervention based on consensus discussions and we will use nominal group techniques to generate ideas for training therapists to deliver the family intervention.

The nominal question is ‘What methods and resources could help support people to effectively deliver family interventions for people with schizophrenia?’

The following steps will guide day 2 workshops:

1. Introductions and presentation of the nominal question
2. Individual time to generate and discuss ideas
3. Round robin feedback from individual group members to record each idea in turn in small group discussions
4. Group discussion of each idea in turn for clarification at the face to face meeting
5. Individual voting on ideas
6. Continue iterative process of feedback and re-ranking until complete

Manual Development

The manual will be developed using the data from these two workshops integrated with an existing evidence-based intervention and will be discussed with the research advisory group.

*Phase 3 Procedures:*

To evaluate the feasibility and acceptability of delivering culturally-adapted FIp to reduce relapse in people with schizophrenia in primary care settings in Java, Indonesia, a rater- blind, randomised feasibility trial will be conducted. Participants will be recruited and selected from among existing service-users with psychotic illness in primary care. Caregivers will be nominated by the service-user or self-refer. Participants will be randomly allocated to ‘culturally-adapted family intervention’ (FIp) or standard care groups, in the ratio 1:1. A schema of participant pathways for both arms is shown in Fig. 1 below.

**CONSORT Flow Diagram**

Numbers analysed

Numbers analysed

Loss to follow-up/discontinuation

Loss to follow-up/discontinuation

Loss to follow-up/discontinuation

Loss to follow-up/discontinuation

Standard care (n= 30)

Family Intervention (n= 30)

Excluded due to ineligibility or declining to participate

## Enrollment

Randomisation (n= 60)

Eligibility screening

## Allocation

## Follow-Up at Completion and 6 Months

## Analysis

*Study Setting and Participant Selection*

This study will be conducted in district *puskesmas* in Bogor and Jakarta. We will recruit up to 10 *puskesmas* with at least one mental health professional providing a mental health programme to people with schizophrenia or other psychotic illness. Like phase 1 recruitment procedures, service-user and carer participants will be recruited via their mental healthcare workers in *puskesmas* or via self-referral in response to our advertisements. Posters and flyers advertising the study will be placed in appropriate locations in *puskesmas* in Bogor and Jakarta such as visiting/relatives’ rooms and noticeboards and community settings such as community centres and carer support groups. Healthcare professionals will be asked if they can identify service-users who may be eligible based on receiving treatment for a diagnosis of schizophrenia or other psychotic illnesses.

Carers will be included if they are

1. Living with or spending at least 10 hours per week in face to face contact with an individual with schizophrenia and assuming a caring role.

2. Carers/Relatives over the age of 18 years.

3. Resident of Jakarta or Bogor

4. Able to give informed written consent

We will exclude potential participants if they have

1. A relative with drug or alcohol dependence alongside a diagnosis of schizophrenia, according to DSM-V criteria.

2. Unstable residential arrangements such that the likelihood of being available for the duration of the trial is low.

Potential participants who express an interest and are capable of consenting (as advised by healthcare workers) will have their contact details forwarded to the research team. The research assistant will post a recruitment pack to the potential participant and will contact them by telephone no earlier than 24 hours after receipt of the pack to invite them to an orientation day in either Bogor or Jakarta. The orientation day will provide an opportunity to give information about the trial process, what the participants can expect in terms of researcher and trial therapist contact, procedures for providing consent and information about trial management. Participants will also have an opportunity to ask questions. Participants will also be given information about the aims and objectives of the trial and asked if they would like to participate in qualitative interviews once the intervention arms are complete. The recruitment pack will contain a participant information sheet and a consent form.

*Randomisation*

Eligible and consenting participants will be randomly allocated to FIp (n=30) or TAU (n=30) via a remote computerised randomisation service. Randomisation will be stratified by regional district (n=2). The Co-PI (HS) will share details of the outcome of randomisation with the individual participants within 48 hours, and with the therapist. Follow-up measurement will be carried out by researcher assistants who will remain blind to treatment allocation.

*Randomised Interventions*

Family Intervention for Psychosis

The intervention is based on a widely used cognitive-behavioural model of FI developed by Barrowclough and Tarrier (1992) and adopted as the model of choice in NICE-approved interventions. The FIp intervention will offer therapeutic elements to enhance family skills and knowledge, communication skills training, enhancing skills in problem-solving and goal setting underpinned by cognitive models and framed by collaborative partnerships to enhance supportive therapeutic relationships. The intervention will be delivered over 12 weeks and will consist of up to 10 sessions. The therapist guide will comprise the manualised intervention guide and details of the intervention will be made explicit following phase 1 and 2.

Treatment as Usual

There will be no restrictions on the care that can be provided as Treatment as Usual (TAU). TAU will be carefully recorded in terms of the timing and nature of any intervention received.

*Feasibility of Recruitment and Intervention Delivery*

We will assess the feasibility of recruiting participants to the intervention using the following outcomes; recruitment numbers = number consented from the number approached, the number of sessions attended by each participant, retention in therapy = the number of participants completed, study attrition = the number who disengaged at each assessment time point and the number who completed assessment measures at study end. We will also retain data on the number who are ineligible at screening and the reasons for not completing at each assessment point i.e. discontinuation/loss to follow-up.

*Assessments*

To identify outcome measures and assess the feasibility of collecting this information in future trials, participants will complete a range of quantitative outcome measures at baseline, post intervention and at the 3-month follow-up. Socio-demographic and contact details will be obtained during eligibility assessment. These measures will be administered by the research assistants at appropriately timed intervals at the *puskesmas* where the service-user and carer are receiving standard care. Research assistants will contact participants in advance to arrange the time of the assessment. Research assistants will operate independent of the therapy. If participants exit early, we will attempt to capture exit information at their exit and at study measurement intervals. Table 1 details the variables of interest, timing of assessments and participants who will are respondents.

Table 1: Variables, Measures and Assessment Points

| *Variable* | *Measure* | *Respondent* | *Eligibility* | *Baseline* | *Post*  *Intervention* | *Three Months Post Intervention* | *Mode of Administration* |
| --- | --- | --- | --- | --- | --- | --- | --- |
| Eligibility | Screen |  | ✅ |  |  |  | Case note review/Self-report |
| Participant age, gender, relationship to service-user, service-user diagnosis, number of hours of contact per week, living status, | Demographic measure | Family member | ✅ | ✅ |  |  | Self-report/Researcher-administered |
| Symptom severity | PANSS | Service-user |  | ✅ | ✅ | ✅ | Researcher-administered |
| Relapse Rates | PANSS | Service-user |  | ✅ | ✅ | ✅ | Researcher-administered |
| Hospital Episodes | Episode count | Service-user |  | ✅ | ✅ | ✅ | Case note review |
| Social Functioning | PSP | Service-user |  | ✅ | ✅ | ✅ | Researcher-administered interview |
| Family Environment and Functioning | FQ | Family member |  | ✅ | ✅ | ✅ | Researcher-supported interview |
| Knowledge, Attitudes, Burden and General Health | GHQ-28, ECI, KAST, IEQ | Family member, Service-user |  | ✅ | ✅ | ✅ | Researcher-supported interview |
| Therapeutic Engagement | Attendance, retention in the intervention, | Trial therapist |  |  | ✅ | ✅ | Researcher-administered interview |
| Fidelity to the Intervention | Fidelity Measure developed in Phase1 and 2 | Trial therapist |  |  | ✅ |  | Researcher-administered interview |
| Acceptability of the intervention | Therapist diaries of number of sessions attended | Trial therapist |  |  | ✅ |  | Self-reported therapist diaries |
| Satisfaction with the intervention | Qualitative interviews | Family member, service-user and trial therapist |  |  | ✅ |  | Qualitative interviews and self-report questionnaires |

*Eligibility Measure*

Screening for eligibility of service-users will be completed by clinician diagnosis and self-report of diagnosis. Caregivers will be eligible if they fulfil previously identified criteria but ordinarily they will be described as a carer by the service-user and may be any relative or significant contact who meets criteria defined.

*Demographic Details*

Sociodemographic details will be collected by self-report will be verified at the start of the assessment by the research assistant.

*Psychosis Symptom Severity, Remission and Relapse*

Clinical symptoms will be evaluated using the PANSS (Kay et al., 1987) which is a valid scale and has been used in many non-English-speaking countries (Ivanova et al., 2018, Hallit et al., 2017, Peralta and Cuesta, 1994). The validity and reliability of the Indonesian version has also been established (Salan et al., 1994). Remission will be determined using the Remission in Schizophrenia Working Group (RSWG) criteria (Andreasen et al., 2005). Relapse will be assessed using the PANSS and we will determine an optimal definition for a future trial based on the varied definitions currently in use. Hospitalisation is the most commonly used proxy measure of relapse but this may not be relevant in an LMIC setting (Olivares et al., 2013). The PANSS will be administered by specially trained research assistants at baseline, immediately after the intervention and 6 months after the intervention.

*Social Functioning*

The Personal and Social Performance Scale (PSP) is a 100-point, observer-rated, single-item scale. The four main areas on which the rating is based include: (a) socially useful activities, including work and study; (b) personal and social relationships; (c) self-care; and (d) disturbing and aggressive behaviours. It is a reliable, acceptable and valid measure of social functioning in people with schizophrenia (Morosini et al., 2000, Patrick et al., 2009). It is sensitive to change in the PANSS scores (Patrick et al., 2009) and has been utilised in Indonesian populations previously (Damanik et al., 2018). Ratings will be made by the research assistant based on service-users reports of symptoms, observations on behaviour during the interview and reports from both referring healthcare workers and caregiver participants. Data will be obtained at baseline, immediately after the intervention and 6 months after the intervention.

*Caregiver Psychological Wellbeing*

The twelve-item General Health Questionnaire (GHQ-12; (Goldberg and Blackwell, 1970)) was developed to screen for non-specific psychiatric morbidity and has been widely validated and found to be reliable. It is commonly used as a screening tool to determine whether an individual is at risk of developing a psychiatric disorder. It comprises 12 likert-type question items that measures a single dimension of psychological health. The Indonesian‐language version of the GHQ‐12 has been tested for reliability and validity, and the Cronbach's alpha ranged from .670 to .776, with a sensitivity of 67.80 and a specificity of 74.75 (Idaiani & Suhardi, 2006).

*Caregiver Burden*

The Involvement Evaluation Questionnaire (IEQ;(van Wijngaarden et al., 2000) is a 31-item questionnaire which is completed by the caregiver. Caregivers who have had more than 10 hours contact with their loved one in the past four weeks will answer a series of questions relating to tension, supervision, worrying and urging and the degree to which the caregiver has experienced any of these. Each question is scored on a 5-point Likert scale. The scale has been developed for European settings, translated and validated in several European countries and in LMIC settings. The scale will be translated into Bahasa following the procedures outlined by Knudsen et al, (2000)) comprising series of translations, back-translations and checking through qualitative inquiry with research assistants and participants in the feasibility study.

The Experience of Caregiving Inventory is a 66 item questionnaire that captures eight negative subscales; difficult behaviours (e.g. him/her being moody, irritable etc); negative symptoms (e.g. him/her being withdrawn etc); stigma (e.g. experiencing stigma of having a mentally ill relative etc); problems with services (e.g. how to deal with mental health professionals etc); effects on the family (e.g. how he/she gets on with other family members etc); loss (e.g. his/her lost opportunities etc); dependency (e.g. being unable to do things you would like etc); need for backup (e.g. having to support him/her etc). There is also two positive subscales; positive personal outcomes (e.g. I have become more understanding of people with problems etc); good aspects of the relationship with the patient (e.g. he/she is good company etc) about the carer’s experiences. Respondents select a rating on a five-point Likert scale from “never” to “nearly always”. Subscale scores and total negative and total positive scores can be obtained by adding up the corresponding subscales. Higher scores on the negative scales indicate greater negative perceptions of caregiving whereas higher scores on the positive scales indicate greater positive perceptions of caregiving. This measure has been used with a variety of carers of mental health conditions. Each subscale has been reported to have satisfactory reliability (Szmukler et al., 1996).

*Family Functioning and Environment*

Expressed emotion (EE) will be measured using the Family Questionnaire (FQ; (Wiedemann et al., 2002). The questionnaire comprises 20 items and each item is measured on a 4-point scale ranging from ‘never/very rarely’ to ‘very often’. The measure consists of two subscales assessing both emotional over-involvement and critical comments. The FQ has excellent psychometric properties including a clear factor structure, good internal consistency of subscales and good inter-rater reliability in relation to the Camberwell Family Interview (CFI; (Vaughn and Leff, 1976) which is the gold standard measure of EE. The FQ also yields consistent significant correlations with the more burdensome, CFI and is similarly accurate and sensitive to predicting components of EE.

*Knowledge*

Knowledge about schizophrenia and psychosis will be measured among participants using the Knowledge About Schizophrenia Test (KAST) (Compton et al., 2007) which was developed for caregivers of people admitted to hospital for treatment of psychosis. The test comprises 21 items regarding the aetiology, onset, symptomatology, outcome and treatment options. The measure shows excellent content validity and good criterion validity. The scale will be translated and adapted for use in Indonesian settings.

*Qualitative interviews (Therapeutic engagement, fidelity, acceptability and satisfaction)*

In-depth interviews will be conducted with participants from both intervention arms at the post-intervention assessment point. We will ask participants about their experiences of the intervention and the trial. All therapists will be required to keep a diary collating quantitative aspects of retention and attendance for feasibility and will be asked also to note their thoughts on participant engagement, delivery of the intervention and barriers to delivery, access and engagement. A sample of therapists delivering the intervention will also be interviewed about their perspectives of the effectiveness and acceptability of the intervention and to explore any barriers to uptake and delivery of the intervention in a full trial. Purposive sampling will be used to select interviewees in order to attempt to capture maximum variation in views and experiences in order that they adequately reflect those of a range of participants. All participants in the trial will be asked if they are willing to be contacted about taking part in a qualitative interview at the time of trial consent. From participants who indicate that they are willing to be contacted, a purposive sample will be drawn in relation to (i) the trial site, (ii) arm of the trial and (iii) socio-demographic variables such as age, gender, ethnicity and socio-economic status. We anticipate sampling will continue until saturation but estimate up to 30 participant interviews and 2-3 therapist interviews. All interviews will be conducted by telephone or face-to-face in a location of the participants’ choice. At interview, a flexible topic guide will be used to ensure primary issues are covered during all interviews, but without dictating data collection, allowing participants to introduce unanticipated issues. Interviews are expected to last between 45-60 minutes. With informed consent, interviews will be recorded using a digital voice recorder, transcribed and anonymized.

*Trial Therapists and Training*

The intervention will be delivered by mental health workers whom we will recruit as trial therapists from the district health centres identified above. Stakeholder workshop participants identified those healthcare workers working to provide the mental health programme in *puskesmas* as the most appropriate individuals to deliver therapy due to their experience, expertise and correspondence between the goals of the mental health programme and the intervention. We will identify *puskesmas* that are resourced with qualified mental health workers in collaboration with the lead for mental health programme delivery in the Ministry for Health. We will recruit and train up to four healthcare workers (non-mental health and mental health workers) in each of two primary care centres at district level to deliver the intervention collaborating with the primary care directorate to conduct research in these sites. Training to deliver the intervention will be delivered by a specialist in caregiver intervention training, Dr Tim Bradshaw. Training for the research aspects of this project will be provided by applicants HS, KL, HB, LR and PB during planned visits to Indonesia for this work.

**IV. DATA COLLECTION**

See attached Data Management Plan

**V. DATA ANALYSIS**

Phase 1 Analysis and Synthesis: We will utilise a six-stage thematic analysis framework using Nvivo software (QSR International Pty Ltd., 2018) to organise text and support analysis. Interviews will be transcribed and independently coded by Indonesian researchers, the coding frame will be translated and verified among the wider research and study team. We will develop an evidence matrix combining empirical findings from this phase of study with empirical findings from existing evidence synthesis of cultural adaptation for psychosocial and mental health interventions. We will draw on two recent systematic reviews; one summarising the content and adaptation process, including moderators and mediators of effective interventions, in meta-analytic studies of culturally-adapted interventions for mental health problems and another meta-analysis and review of culturally-adapted psychosocial interventions specifically for schizophrenia that provides an empirically-derived heuristic framework for cultural adaptation of psychosocial interventions. The synthesis matrix will be developed by the study team tabulating findings from the existing heuristic framework by source of evidence focusing on points of commonality, divergence and representing key ideas about intervention content and delivery, barriers and facilitators to delivery and engagement and supplemented with points about adoption and implementation. The study team have significant experience synthesising evidence for research purposes to identify key intervention components.

Phase 2 Analysis: Using modified nominal group techniques we will determine the groups view on intervention components, manual resources and training needs aggregating responses producing proportional rankings for each component. A minimum defining threshold will be set for accepting items. The manual will be developed by the wider study team defining therapeutic aims and we will develop detailed procedures, patient exercise, materials and resources, good practice examples, scripts for intervention delivery and measures of processes and outcomes.

Phase 3 Analysis:

*Quantitative measures and schedules*

Aggregate quantitative data will be analysed to determine intervention delivery feasibility. Descriptive statistics will be generated to assess attendance, attrition, retention and the completeness of outcome measures. Pre-specified criterion for fidelity will be used interpreting 80%–100% adherence as ‘high’ fidelity, 51%–79% as

‘moderate’ and 0%–50% as ‘low’ fidelity [9]. We will document our intervention according to the template for intervention description and replication (TIDieR) checklist (Hoffmann et al., 2014).

See statistical analysis plan

*Qualitative interviews*

Interview transcripts will be checked for accuracy and then imported into NVivo12 qualitative data analysis software (QSR International Pty Ltd., 2018), to aid management and indexing of data. Thematic analysis (Braun and Clarke, 2006)utilising a data-driven inductive approach will be used to scrutinise the data in order to identify and analyse patterns and themes of particular salience for participants and across the dataset using constant comparison techniques. Analysis will begin shortly after data collection starts, will be ongoing and iterative. Analysis will inform further data collection: for instance, analytic insights from data gathered in earlier interviews will help identify any changes that need to be made to the topic guide during later interviews. Transcripts from the participants and therapists’ interviews will be analysed separately, with coding frames being developed for each. A subset of transcripts will be independently double-coded by other members of the research team and compared and verified among the wider team. Discrepancies will be discussed and resolved to achieve a coding consensus.

**VI. DATA AND SAFETY MONITORING PLAN (if applicable)**

See data management plan attached.

**VII. STUDY LIMITATIONS**

 A. Potential Limitations of Procedures

**VIII. ETHICAL CONSIDERATIONS**

The informed consent process is described above in the section detailing methods. Consent will be obtained from all participants from whom we obtain personal data as per these procedures and we will retain copies of signed consent forms that will be stored securely in both sites. Steps will be taken to minimise coercion; ensuring adequate cooling off period between giving information and requesting consent, allowing the opportunity to ask questions and clarify points and providing contact information for pursuing a complaint. In the first instance, participants may contact Mr Agus Setiawan, Dean for the Faculty of Nursing in Indonesia at Ph. +62 (0) 21 7884 9120 who will implement procedures for handling research complaints at Universitas Indonesia. Dr Herni Susanti and the FUSION research team will be informed by the Dean’s Research Office and Dr Laoise Renwick will ensure that the Research Governance Office at University of Manchester are informed of any events, investigations and outcomes. Ethical information about participant’s rights will be provided at more than one point including first contact, eligibility screening and reiterated at the commencement of data collection.

The key ethical issues that our research raises are how to maintain the confidentiality of participants, protect their anonymity and provide information about our project such that any potential participant can understand the risks of involvement and have an opportunity to decline or consent to participate. We will follow guidelines for International Ethical Guidelines for Health-related Research Involving Humans Council for International Organizations of Medical Sciences (CIOMS) in collaboration with the World Health Organization (WHO). We will obtain individual written consent for all participants and invite different participants for each successive phase to avoid engaging vulnerable groups in both action and implementation phases of the study. It will be made clear to all participants that participation is voluntary and that they can withdraw from the study at any time, without any impact to their care. In some circumstances it may be necessary for us to break confidentiality particularly if we are notified of a current situation where others are being harmed, danger is present or where an unlawful activity is taking place. We will notify the appropriate local authorities (i.e. clinical lead in cases of harm, police in cases of unlawful activity that causes harm or danger).

There is potential for participants and/or researchers to become distressed by participants' disclosures during interviews. All potential participants will be provided with a participant information sheet written to current NRES guidelines and favourably reviewed by relevant ethics committees. They will be given time to consider participation, and a distress protocol, will be available for use.

We will provide training in ethical conduct of research to researchers working on the study and trial therapists in the feasibility study. A detailed protocol will be approved by the relevant ethical committees and provided to researchers and our RAG. We have detailed how the study will satisfy all MRC and University of Manchester's (UoM's) requirements and guidelines on data management, sharing, security and ethics, including the UoM's Research Data Management policy. We will additionally comply with local regulations in Indonesia. Responsibility for study-wide ethical issues, adherence to the protocol, data management, security and quality assurance will be jointly shared by Dr Susanti and Dr Renwick (Co-PIs)

Ethical approval will be sought from Department of Science, Technology and Higher Education department in Jakarta before obtaining approval from the central ethics department. We will concurrently obtain approval from our host institution's ethical committees (University of Manchester).

**IX. PLANS FOR DISSEMINATION OF FINDINGS**

Conference attendance, peer-reviewed publication and community mental health festival for public engagement and dissemination alongside regular, timed social media engagement.

**X. REFERENCES**

XI. APPENDICES

See attached.

Phase 3 Appendices

Appendix 1a Participant Information Sheet

Appendix 1b Participant Information Sheet

Appendix 1c Participant Information Sheet

Appendix 2 Consent Form

Appendix 3 Zoom Participation Guide

Appendix 4 Distress Protocol

Appendix 5 Recruitment Poster

Appendix 6 Qualitative Interview Schedule and Protocol

Appendix 7 Outcome Measures Inventory and Protocol

Statistical Analysis Plan

ANDREASEN, N. C., CARPENTER, W. T., JR., KANE, J. M., LASSER, R. A., MARDER, S. R. & WEINBERGER, D. R. 2005. Remission in schizophrenia: proposed criteria and rationale for consensus. *Am J Psychiatry,* 162**,** 441-9.

BARROWCLOUGH, C. & TARRIER, N. 1992. *Families of schizophrenic patients : cognitive behavioural intervention,* London, Chapman & Hall.

BRAUN, V. & CLARKE, V. 2006. Using thematic analysis in psychology. *Qualitative research in psychology,* 3**,** 77-101.

COMPTON, M. T., QUINTERO, L. & ESTERBERG, M. L. 2007. Assessing knowledge of schizophrenia: development and psychometric properties of a brief, multiple-choice knowledge test for use across various samples. *Psychiatry Res,* 151**,** 87-95.

DAMANIK, R., EFFENDY, E. & CAMELLIA, V. 2018. Correlation among personal, social performance and cognitive impairment in male schizophrenic patient. *IOP Conference Series: Earth and Environmental Science,* 125**,** 012179.

GOLDBERG, D. P. & BLACKWELL, B. 1970. Psychiatric illness in general practice. A detailed study using a new method of case identification. *British medical journal,* 1**,** 439-443.

HALLIT, S., OBEID, S., HADDAD, C., KAZOUR, F. & KAZOUR, G. R. 2017. Validation of the Arabic Version of the PANSS scale among Lebanese schizophrenic patients. *Journal of Psychopathology,* 23**,** 60-66.

HOFFMANN, T. C., GLASZIOU, P. P., BOUTRON, I., MILNE, R., PERERA, R., MOHER, D., ALTMAN, D. G., BARBOUR, V., MACDONALD, H., JOHNSTON, M., LAMB, S. E., DIXON-WOODS, M., MCCULLOCH, P., WYATT, J. C., CHAN, A. W. & MICHIE, S. 2014. Better reporting of interventions: template for intervention description and replication (TIDieR) checklist and guide. *Bmj,* 7.

IVANOVA, E., KHAN, A., LIHARSKA, L., REZNIK, A., KUZMIN, S., KUSHNIR, O., AGARKOV, A., BOKHAN, N., POGORELOVA, T., KHOMENKO, O., CHERNYSHEVA, K., MOROZOVA, M., RUPCHEV, G., LEPILKINA, T., OZORNIN, A., OZORNINA, N., GOVORIN, N., MALAKHOVA, A., HMARA, N., SHYLOVA, O., HRYHORYEU, A., IVANCHIKOVA, N., RAEVSKAYA, I., GUSAK, P., SKUGAREVSKAYA, M. & OPLER, L. A. 2018. Validation of the Russian Version of the Positive and Negative Syndrome Scale (PANSS-Ru) and Normative Data. *Innovations in clinical neuroscience,* 15**,** 32-48.

KAY, S. R., FISZBEIN, A. & OPLER, L. A. 1987. The Positive and Negative Syndrome Scale (PANSS) for Schizophrenia. *Schizophr Bull,* 13**,** 261-276.

KNUDSEN, H. C., VÁZQUEZ-BARQUERO, J. L., WELCHER, B., GAITE, L., BECKER, T., CHISHOLM, D., RUGGERI, M., SCHENE, A. H. & THORNICROFT, G. 2000. Translation and cross-cultural adaptation of outcome measurements for schizophrenia: EPSILON Study 2. *British Journal of Psychiatry,* 177**,** s8-s14.

MOROSINI, P. L., MAGLIANO, L., BRAMBILLA, L., UGOLINI, S. & PIOLI, R. 2000. Development, reliability and acceptability of a new version of the DSM-IV Social and Occupational Functioning Assessment Scale (SOFAS) to assess routine social functioning. *Acta Psychiatr Scand,* 101**,** 323-9.

OLIVARES, J. M., SERMON, J., HEMELS, M. & SCHREINER, A. 2013. Definitions and drivers of relapse in patients with schizophrenia: a systematic literature review. *Annals of general psychiatry,* 12**,** 32-32.

PATRICK, D. L., BURNS, T., MOROSINI, P., ROTHMAN, M., GAGNON, D. D., WILD, D. & ADRIAENSSEN, I. 2009. Reliability, validity and ability to detect change of the clinician-rated Personal and Social Performance scale in patients with acute symptoms of schizophrenia. *Curr Med Res Opin,* 25**,** 325-38.

PERALTA , M. V. & CUESTA, Z. M. J. 1994. Validation of positive and negative symptom scale (PANSS) in a sample of Spanish schizophrenic patients. *Actas Luso Esp Neurol Psiquiatr Cienc Afines,* 22**,** 171-7.

QSR INTERNATIONAL PTY LTD. 2018. NVivo qualitative data analysis software; .

SALAN, R., BUDIMAN, R., BASTAMAN, T. K., YUNIAR, S., DAMPING, C., KUSUMAWARDHANI, A., PURNAMAWATI, Y. & WIDYANTO, S. P. 1994. PANSS di Indonesia—validitas dan reliabilitas. In Pedoman Definisi PANSS. [In Indonesian]. Jakarta, Indonesia: Department of Neuropsychiatry, Faculty of Medicine, Indonesia University.

SZMUKLER, G. I., BURGESS, P., HERRMAN, H., BENSON, A., COLUSA, S. & BLOCH, S. 1996. Caring for relatives with serious mental illness: the development of the Experience of Caregiving Inventory. *Soc Psychiatry Psychiatr Epidemiol,* 31**,** 137-48.

VAN WIJNGAARDEN, B., SCHENE, A. H., KOETER, M., VÁZQUEZ-BARQUERO, J. L., KNUDSEN, H. C., LASALVIA, A. & MCCRONE, P. 2000. Caregiving in schizophrenia: development, internal consistency and reliability of the Involvement Evaluation Questionnaire – European Version: EPSILON Study 4. *British Journal of Psychiatry,* 177**,** s21-s27.

VAUGHN, C. & LEFF, J. 1976. The measurement of expressed emotion in the families of psychiatric patients. *Br J Soc Clin Psychol,* 15**,** 157-65.

WIEDEMANN, G., RAYKI, O., FEINSTEIN, E. & HAHLWEG, K. 2002. The Family Questionnaire: development and validation of a new self-report scale for assessing expressed emotion. *Psychiatry Res,* 109**,** 265-79.
